# Sorption/Desorption and Kinetics of Atrazine, Chlorfenvinphos, Endosulfan Sulfate and Trifluralin on Agro-Industrial and Composted Organic Wastes

**DOI:** 10.3390/toxics10020085

**Published:** 2022-02-14

**Authors:** Raquel Rojas, Guillermo Repetto, José Morillo, José Usero

**Affiliations:** 1 Area of Toxicology, Department of Molecular Biology and Biochemical Engineering, Universidad Pablo de Olavide, Ctra. de Utrera Km. 1, 41013 Seville, Spain; rrojrod@upo.es; 2 Department of Chemical and Environmental Engineering, University of Seville, Camino de los Descubrimientos s/n, 41092 Seville, Spain; jmorillo@us.es (J.M.); usero@us.es (J.U.)

**Keywords:** pesticide removal, organic residues, adsorption isotherms, desorption, kinetic models

## Abstract

The use of pesticides presents a risk to terrestrial and aquatic ecosystems. For this reason, the development of strategies to prevent and restore pollution is of the greatest interest, including the adsorption to organic matter. The aim of the present study was to investigate the sorption/desorption and kinetics of atrazine, chlorfenvinphos, endosulfan sulfate, and trifluralin onto several raw organic wastes by batch experiments. Three kinetic models were used to fit the obtained sorption kinetics data and two to fit the obtained adsorption isotherm data; both the Freundlich and pseudo-second-order kinetic models described the sorption isotherms well. The desorption study revealed hysteresis in all cases, showing strong, and not completely reversible, adsorption in most cases, with the exception of atrazine-sawdust and chlorfenvinphos-sawdust and chicken manure combinations, for which responses were weak and irreversible. The best kinetic, adsorption and desorption constants were achieved for the hydrophobic pesticides. With respect to sorption-desorption rates, orujillo was found to be the best adsorbent for atrazine, while composted urban solid waste was more suitable for trifluralin and endosulfan sulfate. Sorption constants and simple correlations indicated that, not only the organic matter content, but also the nature of the organic matter itself, and the pesticide and adsorbent properties, determine pesticide sorption-desorption. The use of wastes as efficient and cheap adsorbents for reducing the risk of pesticide pollution is proposed.

## 1. Introduction

Although pesticides are widely used in agriculture, there is a clear concern about the contamination of aquatic and terrestrial ecosystems by their residues. A significant proportion of ground and surface water contamination is caused by pesticide leaching on land, and by direct losses during filling and cleaning operations [1]. To meet European environmental quality standards in the field of water policy [2], efforts must be made to prevent groundwater contamination. A possible strategy to reduce the risk of point source contamination by pesticides is to use organic matter as a soil amendment in agricultural practices [3,4,5,6,7,8,9] or to use it in on-farm biopurification systems, commonly referred to as a “biobed” [4,10]. These methods, compared to several techniques used for pesticide removal (e.g., sorption on activated carbon, oxidation with ozone, photocatalytic degradation, etc.), present many advantages, such as being cheaper, as well as being reliable and labor-efficient, with low waste disposal costs. However, there is still a lack of studies evaluating the possible application of locally available, low-cost adsorbents to control or prevent the contamination of water resources caused by pesticides.

The aim of the present investigation is to evaluate the adsorption characteristics of atrazine, endosulfan sulfate, chlorfenvinphos, and trifluralin on different organic matrices (i.e., sawdust, chicken manure, olive oil solid waste—called “orujillo”—and composted urban solid waste). The studied pesticides were chosen based on their physicochemical characteristics, frequency of detection, and toxicity. Numerous studies have reported the contamination of water resources by these chemicals in recent years [11,12,13,14,15,16,17,18]. On many occasions, these levels have been above the 0.1 μg L^−1^ groundwater quality standard for individual pesticides, or 0.5 μg L^−1^ for the sum of several pesticides, which are the current limits allowed under EC Directive 2006/118/EC for the protection of groundwater against pollution and deterioration [19]. Furthermore, several pesticides selected for this study, or their precursors, were catalogued as priority substances under Directive 2013/39/EU [2]. 

The selection of the organic matrices was based on abundance, availability, and cost. Agricultural waste materials have little or no economic value and often pose a disposal problem. Applying them as adsorbents could also help in solving the waste concern and contribute to the development of a circular economy, as many of these wastes come from production processes which are not energy-intensive and, therefore, are quite sustainable [20]. Moreover, these wastes contain nutrients and have other properties that enhance crop production. By this means, the use of chicken manure as a soil amendment results in an increase in microbial respiration [21]. Olive oil solid waste is free of heavy metals and pathogenic microorganisms, and its application significantly increases soil aggregate stability, having great potential for improving the soil structure of coarse-textured soil in the short- or long-term, and increasing the concentrations of nutrients and organic matter, phenolic compounds and total microbial count, so its addition to agricultural soils has been proposed as a solution for its disposal [22,23,24]. The use of wood residues as low-cost adsorbents has been proposed for the immobilization of pesticides in soil and biobeds [4,10]. Using wood residues improves soil properties, such as soil water content and water use efficiency, decreases the bulk density of the soil amended while soil aggregates increase, reduces runoff during heavy rainfall, and increases nitrogen uptake and the crop yield of barley [20]. Composted urban solid waste is increasingly used in agriculture as a soil conditioner (composted materials can contribute to the humic matter of soils) [22], but also as a fertilizer because it usually contains a great variety of nutrients [20]. 

In this context, the adsorption and desorption characterization of the selected low-cost adsorbents could help to prevent water contamination by pesticides. According to the published literature, there is still a lack of information about the sorption of the proposed pesticides onto different organic surfaces, especially for chlorfenvinphos. The adsorption of atrazine, trifluralin, and endosulfan sulfate has been studied more, especially in soils and biochars, but not so far in fresh organic wastes. Thus, the capacity and underlying mechanisms of adsorption of these pesticides by raw organic wastes remains unclear.

To better define the best organic matrices for each proposed pesticide and the sorption-desorption mechanisms, several sorption experiments were performed in this study, including sorption kinetics, sorption isotherms and desorption. In addition, organic wastes were characterized according to pH, specific surface area, and elemental composition to study their possible contribution to adsorption. This characterization is also relevant to be able to compare some amendments with others, bearing in mind that there is still a great lack of information at present [20].

## 2. Materials and Methods

### 2.1. Chemicals and Organic Matrices

All pesticide used in this study (i.e., atrazine, endosulfan sulfate, chlorfenvinphos, and trifluralin) were obtained from Dr. Ehrenstorfer GmbH (Augsburg, Germany). All other solvents and chemicals used were of gas chromatography (GC) or analytical grade and were obtained from Merck (Merck KGaA, Darmstadt, Germany). A Milli-Q water purification system (MilliporeSigma, Burlington, MA, USA) was employed. Sodium chloride and methanol were obtained from Merck (Merck KGaA, Darmstadt, Germany). Working solutions were prepared by diluting the stock solution, first with methanol and then with ultrapure water. The concentration of solvent in the final pesticide solution was less than 0.1%. The standard stock and working solutions were stored at 4 °C and used to prepare dilute solutions and to spike the water samples. Calcium chloride (CaCl_2_) solution (0.01 M), to be used as the solution phase for each batch experiment, was freshly prepared with ultrapure water. 

The pesticides considered belong to different families and their physicochemical properties are shown in Table S1 according to the Pesticide Properties Database [23].

The organic waste substances chosen were collected from different sites in Seville province (south-western Spain). Sawdust (OR1) was provided by a carpenter, chicken manure (OR2) and composted urban solid waste (OR4) were obtained from a farm located in the agricultural village of Trajano, and olive oil solid waste “orujillo” (OR3) was supplied by a local olive oil processing plant. The organic residues were air-dried for a week and then dried at 70 °C in a drying oven for three days. Chicken manure was crushed in an electric mixer and the organic wastes were manually sieved and particle sizes of less than 1 mm were used.

Table 1 summarizes the main physicochemical properties of the organic wastes used. The pH of the organic residues was analyzed using a direct-reading pH meter, according to the usual methodology, and standard procedures from [24] in a 1/2 (*w*/*w*) soil/deionized water mixture. The organic carbon content was determined by an elemental carbon analyzer (TOC-V CHS Total Organic Carbon Analyzer, Shimadzu Corporation, Kioto, Japan). The specific surface area of the organic residues was determined using the Brunauer, Emmett, and Teller (BET) gas adsorption method for dry surface area measurement, based on nitrogen adsorption–desorption isotherms at 77 K within the 0.03 to 0.3 relative pressure range (Micromeritics Gemini Mod 2360 ID:853 with micromeritics Flow Prepo60, Star driver Windows Application). Elemental analysis of the sorbents and soil was performed with EDS (energy dispersive X-ray). The main heavy metals (i.e., Pb, Cu, Hg) were below detection limits. The organic waste samples were measured to confirm that they were free of the pesticides. Data analysis of the pesticide extraction from the organic waste samples showed that pesticide content was under the quantification limits (results not shown).

### 2.2. Sorption Experiments

Sorption of the sorbents was conducted by a batch technique based on previous studies and guides, and according to laboratory conditions and studied substrates [5,25,26,27,28]. Soils take a long time, i.e., days or even months, to attain adsorption or desorption equilibria, but pseudo adsorption equilibrium was attained within 4 h equilibrium studies (see Section 2.2.2). Briefly, in 100 mL conical glass vials, 50 mL pesticide solutions in deionized water (0.01 M CaCl_2_) were continuously stirred in each assay at 150 rpm in a magnetic stirrer at 20 °C (Komet magnetic stirring bars from H + P Labortechnik, Wasserburg (Bodensee), Germany; magnetic stirrer AG POLY 15 “Twister”, from Gerstel GmbH & Co. KG, Mülheim an der Ruhr, Germany). Sorbent dosage, contact time, or pesticide concentration were selected, depending on the effect studied. After stirring, the solution was centrifuged at 5000× *g* for 15 min and the supernatant was recovered and extracted using the stir bar sorptive extraction technique and quantified by GC-MS (See Section 2.3). The aqueous phase used was a CaCl_2_ solution to improve centrifugation and minimize cation exchange [28], to minimize adsorbent mineral balance disruption and maintain constant ionic strength [6,29]. This concentration corresponds to a very low ion strength [30] and it is the usually followed procedure [28,29,30,31,32,33]. According to previous literature, common centrifugation forces used are between 500–5000× *g* [9,25,27]. OECD guideline 106 suggests a high-speed approach is preferable, e.g., centrifugation forces > 3000× *g*, temperature controlled, which is capable of removing particles with a diameter greater than 0.2 µm from aqueous solution [28]. Therefore, a higher centrifugation force of 5000× *g* was used to properly separate the solid and liquid phases; the results were then comparable to studies performed under similar conditions. Visual examination suggested that the sediment and aqueous phases were well separated upon centrifugation; the size of the particles that remained in solution after this centrifugation was assumed to be lower than 0.2 µm—other studies carried out in previous research confirm its validity [25,27,34,35].

#### 2.2.1. Effect of Sorbent Dosage

Several adsorbent weights (0.01, 0.025, 0.05, 0.1, 0.2, 0.3, 0.5, 0.7 and 1 g) were used to investigate pesticide adsorption capacity in 50 mL of pesticide solution (200 μg L^−1^), which was the last water solubility point for all studied pesticides (see Table S1). The mixtures were stirred for 8 h (150 rpm) and treated as previously described (see Section 2.2). Adsorption was calculated based on the difference between the pesticide concentration in the supernatant solution at equilibrium (C_e_) and the pesticide concentration in a reference solution without adsorbent (C_i_).

#### 2.2.2. Kinetic Study

##### Equilibrium Time

Experiments were performed according to Section 2.2. using 50 mL pesticide solutions of 200 µg L^−1^ with 0.6 g of each organic residue continuously stirred at 150 rpm at constant temperature. The concentrations of the pesticides in the solution were analyzed after 5, 10, 20, 30, 40, 50, 60, 90, 120, 240, and 480 min. A sample without organic matrix was subjected to the same conditions.

The amount of analyte adsorbed at any time t, Q_t_ (µg adsorbate/g adsorbent), was calculated using the following mass balance equation:

(1) $$Q_{t}=\frac{C_{i}-C_{t}}{W}\;V$$

where C_i_ and C_t_ (µg L^−1^) are the initial and liquid-phase concentrations of the adsorbate at any time t, respectively, V is the pesticide solution volume (L) and W is the adsorbent mass (g).

All experiments were run in duplicate, with a control of the pesticide solution without organic residue, to account for possible pesticide degradation during the sorption processes.

##### Kinetic Modelling

The concentrations of the pesticides in the solution were determined after 5, 10, 20, 30, 40, 50, 60, 90, 120, 240, and 480 min, so the last point checked was 480 min, one more after equilibrium. This design is in accordance with that applied in previous studies, where sorption kinetics were tested until the same time of equilibrium [36,37,38], or at one [33] or two further points [39]. Three kinetic models were used to fit the experimental data.

The first was the pseudo-first-order kinetic model. The integral form of this model is expressed by the following equation (namely, Lagergren’s equation):

log_10_(Q_e_ − Q_t_) = log_10_Q_e_ − 0.4342 K_1_ t (2)

where Q_e_ and Q_t_ are the quantity of pesticide adsorbed at equilibrium and at time t (µg g^−1^), respectively, t is the time (min) and K_1_ is the equilibrium rate constant of the pseudo-first-order adsorption (min^−1^), which can be determined by plotting log_10_(Q_e_ − Q_t_) versus t. 

The integral form of the pseudo-second-order kinetic model is expressed by the following equation [40]:

(3) $$\frac{t}{Q_{t}}=\frac{1}{K_{2}{\;Q}_{e}^{2}}+\frac{t}{Q_{e}}$$

where K_2_ is the pseudo-second-order kinetic rate constant (g µg^−1^ min^−1^). The value of K_2_ can be determined by plotting t/Q_t_ versus t to obtain a straight line of slope 1/Q_e_ and the intercept of 1/(K_2_ $Q_{e}^{2}$).

The third model was the intraparticle diffusion model. This model, based on the theory proposed by Weber and Morris [41], was tested to identify the mechanistic aspects of sorption. The Morris–Weber equation is:

(4) $$Q_{t}=x_{i}+k_{i}{\;t}^{1/2}$$

where x_i_ is a constant proportional to the boundary layer thickness (µg g^−1^) and k_i_ is the intraparticle diffusion rate constant (µg g^−1^ min^1/2^). The values of k_i_ and x_i_ were obtained from the slope and intercept of the straight line of Q_t_ versus t^1/2^.

#### 2.2.3. Adsorption Isotherms

For the duration of the full analysis (see Section 3.1.2), 4 h was chosen as the equilibrium time for obtaining the adsorption isotherms according to the results of the kinetic study and to achieve a compromise between removal efficiency and experiment duration. 

Adsorption isotherm experiments were carried out by adding 0.6 g of the adsorbents to 50 mL of pesticide solutions of different concentrations (0.005 to 1 mg L^−1^) at room temperature (25 ± 2 °C). The mixtures were agitated in a magnetic stirrer at constant temperature and further treated as previously described in Section 2.2. The adsorption capacity Q_e_ (μg g^−1^) of the adsorbent was calculated using the following equation:

(5) $$Q_{e}=\frac{\left(C_{i}-C_{e}\right)V}{W}$$

where C_i_ and C_e_ are the initial and equilibrium concentrations (μg L^−1^) of pesticide in the liquid phase, respectively, V is the solution volume (L), and W is the mass of dry adsorbent used (g).

Two of the most common models (i.e., Langmuir and Freundlich) were used to fit the experimental data. 

The K_d_ constant was calculated as the ratio between C_s_ (calculated using the Freundlich equation) and C_e_ for a fixed initial concentration, C_i_ = 200 µg L^−1^. The sorption partition coefficient K_d_ is generally related to the fraction of organic carbon associated with the sorbent to yield an organic-carbon partition coefficient, K_oc_, so this constant was calculated (K_oc_ = (K_d_ · 100)/%OC) [6]. 

#### 2.2.4. Desorption

After the adsorption assay, the entire reaction mixture was centrifuged, and the supernatant was carefully decanted. The same amount of decanted supernatant was replaced with 0.01 M CaCl_2_ aqueous solution to maintain the same conditions that applied during the sorption step. The flasks were maintained in a shaker at 150 rpm during 4 h at 25 ± 2 °C. Pseudo adsorption equilibrium was attained within 4 h. Hence, the same time was maintained for desorption studies. After 4 h, an aliquot of samples was taken from the flasks and analyzed. This procedure was repeated three times, resulting in three desorption steps.

### 2.3. Sample Extraction and Analysis

The pesticide removal efficiency was determined by gas chromatography coupled to mass spectrometry using stir bar sorptive extraction. This technique consists in the use of a stir bar (Twister) obtained from Gerstel (GmbH & Co. KG, Mülheim an der Ruhr, Germany) for the adsorption of pesticides in solution (see Section 2.2). The commercial Twister consists of a glass-encapsulated magnetic stir bar 2 cm long, externally coated with polydimethylsiloxane (PDMS). The Twister was placed in an Erlenmeyer flask with 100 mL of the supernatant solution. The extraction was performed for 14 h with a stirring speed of 800 rpm at room temperature (25 ± 2 °C). At the end, the Twister was placed in an empty glass thermal desorption tube (187 × 4 mm i.d.). TD-GC–MS quantification was performed using a Gerstel TDS 2 thermal desorption system equipped with a Gerstel MPS 2 autosampler and a Gerstel CIS 4 programmable temperature vaporization (PTV) inlet in an Agilent 6890 gas chromatograph with a 5973 mass-selective detector (Agilent Technologies). The thermal desorption system was programmed to increase at 60 °C min^−1^ from 40 to 280 °C, and to maintain this temperature for 7 min. Meanwhile, the desorbed analytes were trapped on a liner (Tenax and quartz wool filling) in the CIS 4 PTV injector at 30 °C. Finally, the CIS 4 was adjusted to increase from 30 to 300 °C (held for 7 min) at 12 °C s^−1^ to inject the trapped analytes into the GC column in solvent vent mode. Separation was accomplished on a DB-5 MS fused silica column (29.5 m × 0.25 mm i.d., 0.25 μm film thickness, Agilent Technologies). After the desorption phase, the oven temperature was kept at 70 °C for 2 min and adjusted to rise from 70 to 150 °C at 25 °C min^−1^, then to 200 °C at 3 °C min^−1^, and finally to 260 °C at 8 °C min^−1^. The helium flow (carrier gas) was adjusted using RTL software, such that alachlor was eluted at a retention time of 17.03 min. The mass spectrometer was operated in selected ion monitoring (SIM) mode with electron ionization.

Mass fragments monitored were *m*/*z* 200 as principal ion and then 215, 202, and 173 for atrazine, *m*/*z* principal 272 and then 274, 229, and 237 for endosulfan sulfate, *m*/*z* principal 267 and then 323, 269 and 325 for chlorfenvinphos and *m*/*z* principal 306 and then 264, 307 and 290 for trifluralin. Under these experimental conditions, the retention times were 13.16, 26.69, 21.56, and 11.64 min for atrazine, endosulfan sulfate, chlorfenvinphos and trifluralin, respectively. The amount of pesticide adsorbed is the difference between that initially present in the solution and that remaining after equilibration.

The resulting DLs were 0.001 μg L^−1^ for endosulfan sulfate, 0.003 μg L^−1^ for chlorfenvinphos, and 0.01 μg L^−1^ for atrazine and trifluralin. The relative standard deviations (RSD) were less than 15%, with recovery rates ranging from 94 to 107%.

### 2.4. Statistical Analysis

Pearson coefficient (critical values for two tails) and Student’s *t* analysis were applied to study the correlation among parameters. Excel sheet (version Office 18.2106.12410.0) was used for statistical analysis of the results. Residuals of the regression analysis with the estimated (fitted) values are presented as supplementary data (Tables S2 and S3). 

## 3. Results and Discussion

### 3.1. Sorption Experiments

#### 3.1.1. Effect of Adsorbent Dose

**Figure 1:**
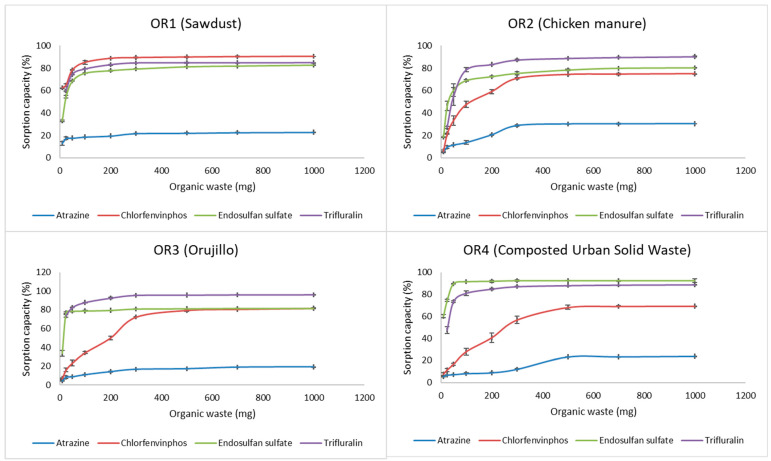
Sorption capacity of the sorbents (OR1—sawdust, OR2—chicken manure, OR3—olive oil solid waste “orujillo” and OR4—composted urban solid waste) for pesticides.

The pesticide adsorption capacity was investigated at several adsorbent dosages (0.01 to 1 g in 50 mL) for 8 h. The sorption capacity (%) of pesticides over the organic matrix was plotted as a function of the adsorbent dose (Figure 1). 

Considering Figure 1, the pesticide removal efficiency increased rapidly when the adsorbent dose was increased from 10 to 100 mg or even by a lesser amount, completing the fast stage at about 100–300 mg. After this, the removal efficiency increased slowly to a maximum. After pseudo-equilibrium, less than 2% variation of pesticide concentration in the solution was observed when adding more adsorbent. To achieve a compromise solution and a general procedure, 600 mg was used in subsequent experiments. Similar results were obtained in previous studies and were explained in terms of saturation of surface sites [42,43]. The soil/solution ratio equivalent to the optimal dose was 1/83. This relation agrees with the one used in similar reports when studying sorption of pesticides on different substrates [25,26,27]. 

In general, the sorption capacity for more hydrophobic pesticides was higher than the sorption capacity of the hydrophilic pesticides, in agreement with a positive relationship between sorption and K_ow_, which was significant for OR2, and an inverse relation with S_w_ (Table S2), except for sawdust, which showed the best adsorption capacity for chlorfenvinphos. Atrazine presented the lowest sorption in all the materials, which may be due to its low hydrophobicity, being the pesticide with the lowest K_ow_ of all those studied (Table S1). 

#### 3.1.2. Kinetic Study

##### Equilibrium Period

**Figure 2:**
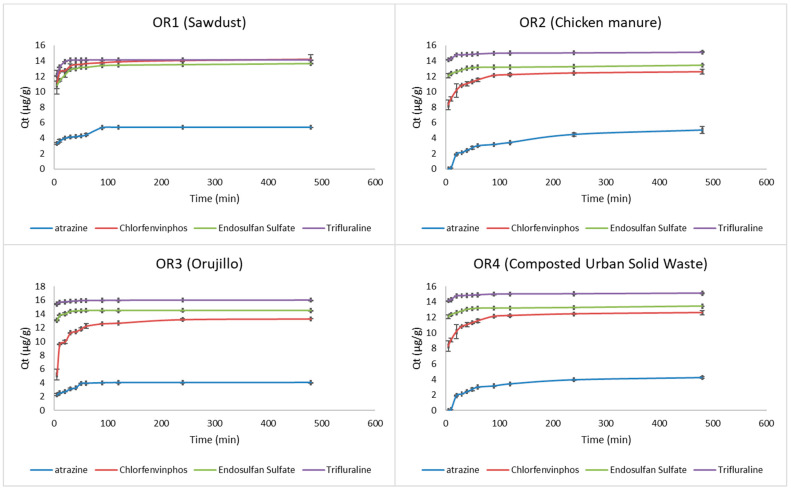
Amount of sorbed pesticides at several times on the four studied organic wastes (OR1—sawdust, OR2—chicken manure, OR3—olive oil solid waste orujillo and OR4—composted urban solid waste).

The measured sorption capacity of each organic matrix for the studied pesticides was plotted as a function of the contact time (Figure 2). When equilibrium conditions are reached, the adsorbate molecules in the solution are in a state of dynamic equilibrium with the molecules adsorbed by the sorbent. After 240 min or pseudo equilibrium, an increase of less than 1% of pesticide concentration in the solution was observed, so 4 h was chosen as the equilibrium period for obtaining the adsorption isotherms. The authors of [35] obtained similar results when studying the adsorption of various pesticides in rice husk ash, with variations between 120–240 min.

##### Kinetic Modelling

Plots were constructed for Lagergren’s and pseudo-second-order models [40] for all studied organic residue/pesticide combinations. The rate constants K_1_ (min^−1^) and K_2_ (g µg^−1^ min^−1^), obtained from the slopes, and the experimental and estimated values of Q_e_ (µg g^−1^) are given with the determination coefficients (r^2^) in Table 2. The time series of adsorbed pesticide concentrations for each pesticide and each substrate were obtained from the batch experiments. The adsorbed concentration after 240 min was assumed to be the equilibrium concentration for the K_1_ and K_2_ calculations. 

The r^2^ coefficients obtained for the pseudo-first-order model data were lower than those obtained for the Ho and McKay model, suggesting that this pseudo-second-order model best describes the adsorption kinetics. Looking at the estimated Q_e_ of each model and comparing it with the experimental Q_e_ (Qe_exp_), a stronger coincidence between Qe_exp_ and Qe_est_ was observed for the second model than for the first. This model has successfully described many adsorption processes, particularly the adsorption of pollutants from aqueous solutions [38], for atrazine when sorbed on biochars obtained from organic wastes [36], for endosulfan sulfate when sorbed on rice husk ash [35], or trifluralin when sorbed on primary polyethylene microplastics [39]. 

A better fit to the pseudo-second kinetic order model suggests that the adsorption rate was controlled by chemical sorption and the sorption capacity was proportional to the number of active sites on the sorbent. Therefore, in general, the controlling mechanism of the adsorption rate was the pesticide adsorption reaction on the adsorbent and not mass transfer. Assessed with the kinetic constant K_2_, the fastest adsorption kinetics were attributed to sawdust for the adsorption of trifluralin, this pesticide being the one that showed the fastest adsorption kinetics for all adsorbents, followed by endosulfan sulfate. Faster and higher sorptions for more hydrophobic compounds were previously obtained by [35] which is supported by a positive correlation between K_2_ and K_ow_ and a negative correlation with S_w_ (Table S2). Pesticides with higher theoretical K_oc_ values (i.e., endosulfan sulfate, trifluralin) (Table S1) were sorbed faster too, suggesting that pesticides strongly sorbed (highest K_d_ or K_oc_) (Table S1 and Table 3) could present a quick sorption process. This is consistent with a positive Pearson correlation between K_2_ and both theoretical (Table S1) and experimental (Table 3) K_oc_ values, which was significant for the organic waste OR2 and OR4 (experimental K_oc_) and OR3 (theoretical K_oc_) (Table S2). 

As mentioned before, OR1 was the adsorbent with the best K_2_ for three of the four studied pesticides, except for endosulfan sulfate, which was better adsorbed by OR3. To provide a first approximation to the influence of the physical-chemical characteristics of the organic residues on the kinetics of the adsorption, simple correlations were applied between the kinetic parameters and the elemental composition of the residues. OR1 stands out because of its high TOC (%) and C (%) content (Table 1). In addition, a positive relationship between TOC or C and K_2_ was found, which was significant for C content and the pesticide trifluralin (Table S3). Furthermore, a significant and negative relationship was observed between K_2_ and Si elemental content (Table S3), OR1 being the studied substrate with the lowest Si content (no Si was detected for this organic waste). An inverse relationship was also found in all cases between K_2_ and the Mg, Cl, and Ca content of the adsorbents; it was notable that none of these elements were detected in OR1, and OR3 showed the lowest concentration of these elements compared to the other three residues. Thus, the presence of these components and elements (i.e., TOC, C, Si, Mg, Cl, Ca) could contribute to the kinetics of the sorption and how fast this process could happen, in agreement with the initial hypothesis that fitting to the pseudo-second-order kinetic model means that pesticide reactions with the adsorbent are the controlling force in adsorption rate. Nevertheless, to check these possible contributions, organic waste should be further characterized, and additional tests applied, such as spectroscopic analyses, to clearly identify the functional groups and the real contribution that these elements may make, as well as direct tests with the addition of such components, as was recently investigated by other authors [44]. However, detailed study of adsorbents is not usually carried out, perhaps because the necessary equipment is not always available [45], but this study and others have highlighted the importance of doing so in order to better interpret the nature of the reactions and to be able to compare the results of different studies, as this may be the key to explaining the wide variety of adsorption behaviors under different conditions. 

Extra theoretical adsorption models are usually implemented to accurately discriminate between adsorption processes, which are usually divided into four steps: (i) transport in bulk solution: mass transfer of solute from solution to the boundary film; (ii) diffusion across the film surrounding the adsorbent particles: mass transfer of solute from boundary film to surface; (iii) diffusion in the pores of the adsorbent: sorption and ion exchange of ions from sites; and, finally, (iv) adsorption on the solid surface or internal diffusion of solute. The intraparticle diffusion model described by the Weber and Morris equation was applied to determine the pesticide adsorption mechanism. According to Equation (4), the plot of Q_t_ versus t^1/2^ should be a straight line with a slope k_i_ and intercept at x_i_, when the adsorption mechanism follows the intraparticle diffusion process. The value of x_i_ gives an idea of the thickness of the boundary layer, i.e., the larger the intercept, the greater is the boundary layer effect [38]. The determination coefficients for the intraparticle diffusion model included in Table 2 show that the adsorption plot was not linear over the whole time range for most of the studied pesticides. This is supported by the plots of the Morris–Weber equation for the pesticides’ average adsorption (see Figure S1) where, in most of the cases, at least two different stages could be observed: external mass transfer (defined by x_i_) followed by intraparticle diffusion (defined by K_i_) [46], with, finally, the balancing stage being reached. The plot of Q_t_ versus t^1/2^ was typically multilinear, which implies that two or more steps occur in the adsorption processes [37] and indicates that intraparticle diffusion is involved in the adsorption process, but that it is not the only rate-limiting step [35]. 

The boundary layer effect (higher x_i_) was greater for endosulfan sulfate and trifluralin when sorbed on all organic residues (Table 2). This is in keeping with their higher molecular weights and with their lower water solubility, suggesting a positive relation between film diffusion and molecular size, which was supported by an inverse relationship between film diffusion and the water solubility of the studied pesticides (Table S2). In contrast, atrazine presented the lowest x_i_ values, so it spread easily. Regarding the amendments, OR4 was the substrate with the lowest x_i_ values for the studied pesticides. This organic waste was the one with higher pH, specific surface area (BET), and Mg, Si, Cl, and Ca content (Table 1). A negative and, in some cases, significant relation between pH, BET, Mg, Cl, Si and Ca content and x_i_ was observed (Table S3), so a possible role of these parameters in the external diffusion through the boundary layer and on sorption is suggested; it would be interesting to confirm this in future research with direct and specific experiments.

After the complexation of the film diffusion layer step, the pesticide molecules are transported from the surface of the adsorbent and along the pores by intraparticle diffusion. The constant K_i_ indicates the degree to which this stage occurs. The highest K_i_ values were obtained for the pesticide chlorfenvinphos, which was the most water-soluble. In general, the highest K_i_ values were obtained for more polar pesticides (i.e., those with highest water solubility and lower k_ow_). The above is in accordance with the observed correlation coefficients between K_i_ and S_w_, which were positive in all cases and even significant for orujillo (OR3) (Table S2). In contrast, this Pearson coefficient was negative when K_i_ was correlated with the water octanol coefficient (Table S2). 

#### 3.1.3. Adsorption Isotherms and Adsorption Model 

The values of the Freundlich and Langmuir model constants and the values of the determination coefficient r^2^ for the four studied organic residues are shown in Table 3.

The values of the determination coefficient for almost all cases were quite high and similar between the Freundlich and Langmuir models. For endosulfan sulfate on OR1 and OR2, and for chlorfenvinphos and atrazine on OR2, the Langmuir model was not applicable, as negative values for the Langmuir constants Q_m_ and K were obtained, which is improbable. This may indicate that monolayer adsorption assumed in this model was not valid for these specific experiments [1,5] and that the Langmuir model was not applicable for the adsorbent OR2 for three of the four studied pesticides. Although the experimental results show that, in most of the studied cases, both models can describe the adsorption process, the Freundlich model is applicable in all cases.

Comparison of the sorption capacities of different sorbents based on K_f_ values is only possible when they show similar n_f_ values [47]. Therefore, based on the results (Table 3), it was not possible to carry out a precise comparison of sorptivity using K_f_ values, so K_d_ values were used to compare the sorption capacity of the studied sorbents. Endosulfan sulfate and trifluralin sorption constants obtained for all studied organic matrices were higher than those observed for more polar compounds, supported by a positive Pearson coefficient between K_d_ and K_ow_, and a negative one between S_w_ and K_d_ (Table S2), in agreement with the results obtained by other authors [9,35]. It is worth noting that the same trend was observed with respect to the K_f_ and K_oc_ values, and the same tendency is visually reflected when representing Freundlich sorption isotherms (Figure S2). 

K_oc_ values were also calculated from the fit of the experimental sorption isotherms. OR1 and OR3 showed a similar %TOC (about 50%), while OR4 and OR2 contained 25 and 18 %TOC, respectively (OR1 ≈ OR3 > OR4 > OR2). However, in the case of endosulfan sulfate, chlorfenvinphos, and trifluralin, K_oc_ sorption followed a decreasing order of OR4 > OR3 > OR2 > OR1. K_oc_ values for atrazine were in the order of OR2 > OR3 > OR4 > OR1, despite OR1 being the best adsorbent for atrazine considering k_d_. Therefore, the sorption on these substrates cannot be attributed only to their organic carbon content, which agrees with a negative correlation between K_oc_ and %TOC, as well as with C (Table S3). Weak or negative correlations between sorption parameters and TOC support the previous suggestions (Table S3). Similar results were observed by [48] and [36]. Other factors, such as the nature of the organic matter or physicochemical characteristics of the surface could be involved. The authors of [49] explained that pesticides with different hydrophobic grades could be adsorbed by partition of compounds with hydrophobic units of phenylpropane and lignin. This is in line with other studies, some of which include triazines, according to the review by [20]. This could explain the good sorption obtained for atrazine when using OR1 or OR2, as these substrates are rich in these compounds [35,49]. Moreover, it is widely recognized that chemical sorption is also affected by the quality or nature of the organic matter [1,6,31]. OR4 is a composted waste, and therefore it is more likely to have organic matter with a certain level of humification than the other adsorbents. This may explain why it was demonstrated to be the best adsorbent for endosulfan sulfate, chlorfenvinphos, and trifluralin. The possible contribution of the different properties of the organic wastes was studied by simple correlation (Table S3). The use of linear regression as a tool for predicting pesticide sorption-desorption was proposed in [50], at least as a first approximation. A positive correlation was observed between K_d_, K_f_, or K_oc_ values and pH or BET, with the exception of atrazine. The last observation is in agreement with the results obtained by [36], who explained that negative correlations with pH indicated that the lack of deprotonation at lower pH may be favorable for the adsorption of the ionizable compounds. According to [20], numerous studies also found that, in general, adsorption was greater the larger the specific surface area, while the mechanism and degree of adsorption was at least related to the physico-chemical characteristics of pesticides. In [29], it was also suggested that, apart from specific surface area, the rich surface functional groups, aromaticity, and negative surface charge of adsorbents may increase the sorption capacity via different bindings and interactions. Following these observations, it is worth noting that the specific surface of the residues studied was between 0.25 y 1.60 m^2^ g^−1^ (Table 1), which contrasts with the high specific surfaces that are usually present in other common adsorbents, such as acid-treated adsorbents (average values of 300 m^2^ g^−1^) [5], biochars (>100 m^2^ g^−1^ and usually >1000 m^2^ g^−1^) [29,36,39], or activated carbon (>1000 m^2^ g^−1^) [51]. However, for natural adsorbent materials, they rarely have specific surface areas in excess of 50 m^2^ g^−1^, probably because they are macroporous materials. Nonetheless, by most often having a great variety and quantity of functional groups, this apparent limitation is substantially compensated for and results in good fixatives of various chemical compounds [51]. The specific surface results obtained in this study are similar to those obtained by [49] for sawdust, with specific surface area < 1 m^2^ g^−1^. Analyzing the Pearson’s correlation coefficients between these adsorption parameters and the elemental content, a trend was observed in that the adsorption capacity was positively correlated with the content of Cl, Ca, and Mg (OR4 stands out in terms of the content of these components; Table 1), while the relationship was inverse with the content of K, except for the pesticide atrazine (atrazine is best sorbed in OR1, which has the lowest content in K). This agrees with the results obtained by [52], who found that the addition of calcium significantly increased the sorption of endosulfan sulfate in humic acids. 

It has also been suggested in [31] that the surface mineral composition or physicochemical properties of the adsorbent could contribute to the sorption. However, the correlations obtained with the elements are not significant or weak (*n* = 4, *p* < 0.05), except for the pH parameter in the adsorption of chlorfenvinphos (Pearson = 0.98, *n* = 4, *p* < 0.05) (Table S3). Thus, in this case, and based on the correlations obtained, it seems that the specific surface area or pH could play a role in sorption, while the presence of specific ions is not as clearly influenced by kinetics (see Section 3.1.2—Kinetic Modeling). These possible relations should be interpreted cautiously, and as previously commented, contrasted with other analytical techniques and tests. 

In the present investigation, atrazine showed the lowest sorption rates in almost all the experiments (i.e., influence of adsorbent dosage, equilibrium time, kinetics and adsorption isotherms). This has been explained focusing mainly on its water solubility and low hydrophobicity, but it would be interesting to study the influence of the ionic force in this particular case because, depending on whether the ionic force influences positively or negatively, the adsorption capacity of atrazine on organic residues could be increased by using more or less saline water. In general, it is expected that the adsorption of more polar compounds, such as atrazine, will be favored with the ionic force, since this reduces its solubility in water by decreasing the polarity of the sample. In contrast, those more apolar pesticides, such as trifluralin or endosulfan sulfate, are expected to be diminished for high ionic forces, since the decrease in polarity reduces the equilibrium constant between the aqueous phase and the organic waste and, therefore, the affinity between apolar pesticides and the adsorbent (greater than 10% salt concentration *w*/*v*) [53,54,55]. However, this general trend is not always strictly observed. In the case of atrazine, it sometimes increases with ionic strength, such as when using heat-treated Karolite as an adsorbent [56] or with different inorganic components of the soil ([57], and at other times decreases, as was observed by [58,59] in humic acid (HA)-coated nanoparticles and on carbon nanotubes, respectively. The adsorption of trifluralin increases with ionic strength when a polymer resin is used as an adsorbent, sometimes leading to saturation of the resin surface [30]; the authors explain that this may be due to a decrease in repulsive forces at the adsorbate-adsorbent interface and between the pesticide molecules themselves, since this herbicide has a polar ring that is part of its structure. Conversely, when used as a chitosan adsorbent, trifluralin sorption at high concentrations decreases with ionic strength, due to decrease in the coulombic attraction between the dipole of the trifluralin molecule and the ammonium group of chitosan [45]. The increase in ionic strength from 0.001 to 0.01 M decreases the adsorption of endosulfan sulfate in Standard Elliot soil humic acid [52]. The same happens with alpha endosulfan, when considering its adsorption on humic acids, since its solubility increases, while for beta endosulfan no significant change in adsorption is observed; however, when studying the adsorption of these compounds in fulvic acids, it was observed that the ionic force did not affect the adsorption. For the organophosphate, chlorfenvinphos, it seems that ionic force does not influence the adsorption of this pesticide [59], probably due to its relatively high or medium polarity (K_ow_ 3.85, Table S1). Therefore, ion strength also plays a role in pesticide adsorption/desorption; the positive or negative contribution depends on the pesticide properties but also on the characteristics of the amendment, making it not always easy to predict the relationship, even at low ionic forces. It would be interesting, or even necessary, to carefully study the effect of ionic strength in sorption to gain a better understanding of the sorption processes of the studied pesticides in a particular adsorbent.

Compared with other studies, and in general terms, the K_fa_, k_d_, or K_oc_ values are in line with those obtained for the same pesticides on different sorbents. For example, when studying chlorfenvinphos sorption on soil, soil amended with cow manure, pig slurry or compost or green manure, ref. [60] obtained k_d_ values between 1.9 (soil alone) and 12.4 L Kg^−1^ (soil amended with cow manure). K_oc_ values obtained by [9] for the sorption of chlorfenvinphos on soil amended with ecological composted sheep manure or coir (lixiviation studies) were between 204–322 L Kg^−1^. The authors of [61] studied the sorption of atrazine and trifluralin on soils, obtaining K_fa_ values from 0.9 to 13.0 L Kg^−1^ for atrazine and from 3.9 to 8.8 L Kg^−1^ for trifluralin. Values of trifluralin’s K_fa_ in soil varied from 20.1 to 743 L Kg^−1^ [48,50], and from 290 and 1590 for zeolites and 3240 for activated carbon [62]. In [63], k_d_ values between 106 and 294 were obtained for the adsorption of trifluralin in five agricultural soils of the UK and K_oc_ values of 63 ± 10. k_d_ values of 1.28, 1.6, 57.6, 332 to 807, and 87 to 1010 L Kg^−1^ were obtained for atrazine sorption on ultisol [64], soil amended with sewage sludge [32], soil amended with organic waste from sugar mills [65], biochars from peanut shell [29] and slow pyrolysis biochars from vegetal materials [36], respectively. The adsorption of endosulfan sulfate in clay or composted soils was studied in [66], with obtained K_fa_ values between 200 and 520 L Kg^−1^ and 80 for sandy soil. k_d_ values obtained for endosulfan sulfate in other studies varied between 98 (rice husk hush) [35], 541–834 (sal wood charcoal) [67] and 1500 L Kg^−1^ (composted cotton gin trash) [68]. Thus, the adsorption values obtained in the present study (Table 3) are in line with those previously obtained by other authors, despite the use of modified organic waste, or even biochars, instead of raw materials, in many of them, which shows the high potential of the proposed use of organic amendments as an ecological barrier to prevent groundwater contamination by these pesticides. 

#### 3.1.4. Desorption 

The sorption-desorption experimental results were analyzed using the empirical Freundlich equation ($Q_{e}=K_{f}\cdot {\;C}_{e}{}^{1/n}$), Q_e_ (µg g^−1^) being the amount of pesticide sorbed at the equilibrium concentration, C_e_ (mg L^−1^) the pesticide solution concentration after each desorption step, K_fd_ the Freundlich desorption coefficient (L·Kg^−1^) and n_fd_ a constant which depends on the sorbate, the sorbent and temperature properties. The obtained Freundlich coefficients, K_fd_ and n_fd_, the determination coefficients (r^2^), the hysteresis coefficient (H), and desorption rate (%D) are indicated in Table 4.

The hysteresis coefficient H (=n_fa_/n_fd_ ∙ 100) is a measure of the extent of hysteresis in desorption [36]. In the present study, H values were calculated from n_fa_ and n_fd_ values corresponding to pesticide concentrations up to 200 µg L^−1^, which was the last common water solubility point. A value of 100 means that desorption proceeds as fast as adsorption does and no hysteresis occurs. The adsorption-desorption of all cases in the studio showed hysteresis, in agreement with other studies [50,68,69]. With the exception of chlorfenvinphos, the H values obtained were very high (258 to 4201, Table 4). This agrees with that observed for the K_fd_ and K_fa_ values. It was observed that K_fd_ values were higher than K_fa_ (Table 4), demonstrating that the rate of sorption is higher than that for desorption; thus, it can be said that the sorption is strong and not a completely reversible process [70,71], with H values fewer than 100, or not able to be calculated, coinciding with the highest rates of desorption (100%). This was the case for the pesticide chlorfenvinphos, implying that the sorption of this organophosphate compound is weak and reversible. As previously stated, to obtain a better understanding of the main reactions, bonds and forces that contribute to the high desorption of chlorfenvinphos, in future research, it would be interesting to investigate in more depth the adsorbent characteristics by means of additional tests.

Hysteresis can occur due to the irreversible binding of solutes with the organic carbon of the adsorbent, or by the entrapment of molecules in meso and micropores within the mineral structures of adsorbents [36]. The interpretations made in the present study for H values are supported by the representation of the sorption-desorption isotherms (Figures S3–S6), where all desorption curves were above sorption (positive hysteresis), except for chlorfenvinphos, when OR1, OR3 or OR4 were used as adsorbents (negative hysteresis), and atrazine on OR4. Negative hysteresis has previously been described by other authors in the desorption of other hydrophilic compounds, such as atrazine and simazine [3,72]. Chlorfenvinphos was the pesticide with the highest water solubility of those studied (Table S1)—this parameter was positively related to %D and negatively to K_fd_ and H; moreover, in the case of %D, the relationship was also significant in almost all cases (Table S2). According to the above, in general terms, the adsorption/desorption behavior follows the same trends based on the parameters k_fd_ and %D. The pesticides are in the following order in terms of their ability to be retained in the studied adsorbents: trifluralin > endosulfan sulfate > atrazine > chlorfenvinphos. This is in agreement with the results obtained by [63], who obtained a low desorption rate for trifluralin (3.9–15.2%) while the other pesticide studied, isoproturon, of greater solubility, was desorbed in high percentages (69.7–101.8%). The authors of [50] also obtained greater desorption in soils for hydrophilic pesticides (91.1% for atrazine) but lower for hydrophobic trifluralin (17.3%). They explained this as a result of the high-water solubility and the weak interaction between the pesticide and soil components (e.g., organic matter, clay particles). In addition, [33] reached similar results. Previous observations are supported by a direct relationship (significant in most of the cases) between %D and S_w_, and an inverse one with K_ow_, observed in this study (Table S2). 

An exception to the ordering listed above was observed for OR3, for which the %D for endosulfan sulfate was slightly higher than for atrazine. Desorption of hydrophobic compounds has previously been observed and explained as being due to the presence of dissolved organic matter [71]. Additionally, OR3 is a pomace, and it may contain high hydrophobic oilseed compounds that may have detached and dragged this particular hydrophobic pesticide on them during the desorption process. The specific reason why it influenced endosulfan sulfate but not trifluralin is still unknown, although it could be attributed to components of the adsorbent that may form strong bonds and prevent such desorption.

Furthermore, OR3 and OR4 stand out, showing similar retention capacities when they adsorbed the hydrophobic pesticides trifluralin and endosulfan sulfate, while in the case of atrazine, OR3 was the best adsorbent in terms of retention capacity by far (H values obtained by OR3 were three to almost seven times greater than for the other organic wastes) (Table 4). To discern the possible contribution of the organic waste composition in desorption, simple correlations were calculated between the studied characteristics of the adsorbents and desorption parameters. Orujillo (OR3) stands out above the other studied residues for its high content in K and O, as well as for its low specific surface area (BET) and its low content of Mg, Si, Cl, and Ca. Correlating H with these parameters, a direct relationship with the K and O content was obtained for the pesticide atrazine, being significant for the O content. Negative correlation coefficients were obtained for the coefficient for hysteresis, and the coefficients for atrazine and Mg, Si, Cl, and Ca content, being significant in the case of Si. The correlation with BET was also negative. Similar, and analogous, results were obtained by correlating with K_fd_ or %D (Table S3). This could explain the relatively high retention potential of OR3 for atrazine. In contrast, the positive correlation obtained for Cl and Ca with K_fd_ and H (or the negative one for %D) for both hydrophobic pesticides, endosulfan sulfate and trifluralin, indicates that these elements could favor the retention of these pesticides (OR4, which was as good or better an adsorbent in terms of retention capacity for trifluralin and endosulfan sulfate, standing out precisely for its high content in Mg, Si, Cl and Ca (Table 1)). The same was true for Mg and Si in the case of endosulfan sulfate. However, it is worth noting that trifluralin sometimes behaved like atrazine and sometimes like endosulfan sulfate and no significant relations were found in these cases, so it could be said that these elements do not play a decisive role in the retention of this pesticide, and that other factors must intervene. Chlorfenvinphos was completely desorbed in all cases. When the analysis was carried out, based on K_fd_ and %D, similar results were obtained. 

Thus, desorption appeared to be the result of a combination of several chemical and physical processes. In this study, it has been proposed that there are possible contributions of organic waste composition on desorption, based on simple correlations. It would be of great interest to check these results with more detailed studies, focusing on the desorption process and the factors affecting this, which would allow identification and separation of the physical and chemical processes involved.

## 4. Conclusions

The present investigation examined the sorption-desorption and kinetics of four pesticides in several organic wastes. 

Under the experimental conditions, 600 mg of the studied adsorbents (sawdust, orujillo, chicken manure and composted urban solid waste) were enough to remove pesticides in solution and 4 h was sufficient time to reach equilibrium.

A kinetic evaluation of the equilibrium data suggested that the pseudo-second-order model best described the adsorption kinetics. Consequently, the sorption rate for these pesticides was controlled by chemical sorption rather than mass transfer. Faster kinetic sorption was observed for hydrophobic compounds, it being notable that, in general, pesticides with higher adsorption rates were those that were adsorbed more quickly. Pesticides adsorbed quickly on orujillo, with the exception of trifluralin, which was more rapidly adsorbed by sawdust; composted urban solid waste was the organic waste with the lowest kinetic constants in every case. 

Sorption isotherms fitted the Freundlich model, whereas the Langmuir model was not appropriate. The results revealed that sawdust, chicken manure, orujillo, and composted solid urban waste could be effectively used to remove atrazine, endosulfan sulfate, chlorfenvinphos, and trifluralin, with adsorption rate values similar to those previously obtained by other authors. This was despite the use of modified organic waste or even biochars instead of raw materials in many of them, which shows the high potential of the proposed use of organic amendments as an ecological barrier to prevent groundwater contamination by these pesticides. Composted urban solid waste was shown to be the best adsorbent for chlorfenvinphos, endosulfan sulfate and trifluralin, followed by orujillo; sawdust and chicken manure were shown to be more suitable for atrazine. 

The adsorption-desorption studies showed hysteresis. In most cases, adsorption was strong and irreversible, except for chlorfenvinphos, which showed negative hysteresis. Desorption was positively related to pesticide water solubility and inversely to the octanol-water coefficient. Orujillo was the organic waste with the highest retention capacity for atrazine and trifluralin, with at least 85% of the previously adsorbed pesticide retained, while composted urban solid waste showed the highest retention capacity for the organochlorine, endosulfan sulfate (81% retention rate). 

Considering both the rate of adsorption and of desorption, and if the purpose is to use organic waste as an amendment, orujillo is a good adsorbent for atrazine. For trifluralin and endosulfan sulfate, composted urban solid waste is the recommended adsorbent, closely followed by orujillo. Care should be taken with chlorfenvinphos due to its high desorption rate.

It can be concluded that adsorption, desorption and kinetic processes are influenced not just by one factor, but by the confluence of several, such as the organic matter content and composition and the physicochemical characteristics of the pesticides and adsorbents. For a better understanding of the sorption process for a particular pesticide onto a determined substrate, it is crucial to characterize, as far as possible, the organic waste and the contact solution, something that is beginning to be done, although not always. This would enable future studies to be carried out that address this aspect in more depth, to better understand the relationship between the variables, and even to create adsorption-desorption prediction models based on the physical and chemical characteristics of pesticides and adsorbents.

Finally, based on the results, as a general conclusion, it could be stated that the studied organic wastes are of possible use as effective adsorbents for pesticides, because of their high adsorption capacity, while also being natural and of low cost.

## Figures and Tables

**Table 1:** Physicochemical properties of the studied amendments.

	TOC	SE	pH	C	N	O	Na	Mg	Al	Si	P	S	Cl	K	Ca	Ti	Fe
	%	(m^2^ g^−1^)	upH	%	%	%	%	%	%	%	%	%	%	%	%	%	%
OR1	50.0	0.43	6.45	68.0	-	32.0	-	-	-	-	-	-	-	-	-	-	-
OR2	17.9	0.85	7.06	53.4	1.60	33.2	0.10	0.50	0.80	2.70	0.30	0.20	0.50	1.80	3.40	-	1.20
OR3	48.9	0.25	7.04	56.3	2.40	37.4	-	0.40	-	0.30	-	-	0.20	2.40	0.60	-	-
OR4	25.4	1.60	7.53	50.1	-	30.2	1.40	0.60	1.30	3.10	-	1.30	1.00	0.90	8.20	0.10	1.80

Footnotes: OR1 (sawdust), OR2 (chicken manure), OR3 (olive oil solid waste, orujillo), OR4 (composted urban solid waste). SE: surface specific area. TOC: total carbon content. SE: superficial area (m^2^g^−1^) obtained by BET (The Brunauer, Emmett and Teller gas adsorption method for dry surface area measurement).

**Table 2:** Experimental and estimated values of the studied kinetic models.

			Lagergren			Ho and McKay			Morris-Weber		
	Pesticide	Qe_exp_	Qe_est_	K_1_	r^2^	Qe_est_	K_2_	r^2^	k_i_	x_i_	r^2^
OR1	Atrazine	5.38	1.98 ± 0.06	0.013 ± 0.001	0.92	5.49 ± 0.01	0.024 ± 0.002	1.00	0.111 ± 0.006	3.55 ± 0.10	0.72
	Chlorfenvinphos	14.0	2.20 ± 0.71	0.024 ± 0.008	0.78	14.9 ± 0.05	0.014 ± 0.002	1.00	0.197 ± 0.063	11.4 ± 1.50	0.43
	Endosulfan sulfate	13.5	2.25 ± 0.44	0.031 ± 0.005	0.96	13.7 ± 0.04	0.036 ± 0.001	1.00	0.115 ± 0.012	11.8 ± 0.21	0.54
	Trifluralin	14.1	0.05 ± 0.00	0.023 ± 0.001	0.97	14.1 ± 0.00	1.442 ± 0.047	1.00	0.002 ± 0.000	14.1 ± 0.00	0.67
OR2	Atrazine	4.46	3.79 ± 0.12	0.012 ± 0.001	0.86	5.51 ± 2.39	0.005 ± 0.000	0.99	0.245 ± 0.020	0.47 ± 0.07	0.82
	Chlorfenvinphos	12.4	4.75 ± 0.85	0.030 ± 0.002	0.97	13.3 ± 0.98	0.011 ± 0.003	1.00	0.260 ± 0.041	8.68 ± 0.97	0.60
	Endosulfan sulfate	13.3	0.92 ± 0.14	0.027 ± 0.001	0.84	13.5 ± 0.00	0.053 ± 0.003	1.00	0.059 ± 0.007	12.4 ± 0.10	0.63
	Trifluralin	15.0	0.78 ± 0.01	0.031 ± 0.001	0.96	15.1 ± 0.06	0.089 ± 0.024	1.00	0.040 ± 0.002	14.4 ± 0.00	0.56
OR3	Atrazine	4.03	3.13 ± 0.09	0.054 ± 0.002	0.93	4.09 ± 0.01	0.046 ± 0.003	1.00	0.088 ± 0.005	2.68 ± 0.08	0.55
	Chlorfenvinphos	13.2	4.56 ± 0.79	0.021 ± 0.003	0.91	13.5 ± 0.03	0.013 ± 0.002	1.00	0.257 ± 0.059	9.12 ± 0.85	0.58
	Endosulfan sulfate	14.5	2.12 ± 0.06	0.088 ± 0.009	0.99	14.5 ± 0.00	0.219 ± 0.054	1.00	0.046 ± 0.005	13.8 ± 0.08	0.35
	Trifluralin	16.0	0.41 ± 0.02	0.030 ± 0.002	0.94	16.0 ± 0.00	0.232 ± 0.021	1.00	0.021 ± 0.002	15.7 ± 0.03	0.51
OR4	Atrazine	3.79	4.15 ± 1.09	0.010 ± 0.003	0.89	4,85 ± 0,15	0,003 ± 0.00	0.97	0.233 ± 0.009	-0.43 ± 0.06	0.87
	Chlorfenvinphos	14.6	9.09 ± 1.55	0.036 ± 0.006	0.85	13.8 ± 0.16	0.004 ± 0.001	1.00	0.549 ± 0.030	4.24 ± 0.53	0.54
	Endosulfan sulfate	11.9	2.57 ± 0.88	0.030 ± 0.009	0.69	16.1 ± 0.27	0.004 ± 0.001	0.95	0.330 ± 0.021	10.2 ± 0.32	0.22
	Trifluralin	16.0	2.14 ± 0.51	0.058 ± 0.003	0.86	16.1 ± 0.03	0.047 ± 0.016	1.00	0.200 ± 0.037	13.3 ± 0.51	0.19

Footnotes: K_1_ (min^−1^): Lagergren kinetic constant. K_2_ (g µg^−1^ min^−1^): Ho and McKay rate constants. x_i_ (µg g^−^^1^): Morris–Weber constant, proportional to the boundary layer thickness)_._ k_i_ (µg g^−1^ min^1/2^): intraparticle diffusion rate constant. r^2^: determination coefficient (r^2^) of the studied kinetic models. (OR1 = sawdust, OR2 = chicken manure, OR3 = olive oil solid waste orujillo, OR4 = composted urban solid waste).

**Table 3:** Freundlich and Langmuir parameters obtained for different adsorbents. (OR1 = sawdust, OR2 = chicken manure, OR3 = orujillo, OR4 = composted urban solid waste).

		Freundlich			Langmuir			k_d_	K_oc_
		n_f_	K_f_ (L kg^−1^)	r^2^	Q_m_	K	r^2^	(L kg^−1^)	(L kg^−1^)
OR1	Atrazine	0.77 ± 0.06	67.7 ±21.2	0.94	1.73 ± 0.57	0.050 ± 0.017	0.84	39.8 ± 7.63	79.5 ± 15.2
	Chlorfenvinphos	0.63 ± 0.10	189 ± 46	0.88	3.43 ± 0.95	0.036 ± 0.012	0.90	33.4 ± 2.82	66.7 ± 5.65
	Endosulfan sulfate	0.83 ± 0.02	753 ± 80	0.96	−9.72 ± 3.72	−0.051 ± 0.020	0.95	358 ± 65.9	715 ± 132
	Trifluralin	0.93 ± 0.05	571 ± 146	0.99	16.3 ± 3.76	0.038 ± 0.004	1.00	461 ± 0	922 ± 0
OR2	Atrazine	0.79 ± 0.10	136 ± 34	0.84	−3.98 ± 0.39	−0.012 ± 0.001	0.65	30.5 ± 2.3	169 ± 13
	Chlorfenvinphos	1.12 ± 0.08	15.6 ± 4.0	0.97	−1.91 ± 0.04	−0.008 ± 0.002	0.97	35.3 ± 0.48	197 ± 3
	Endosulfan sulfate	0.91 ± 0.01	493 ± 53	0.99	−562 ± 297	−0.001 ± 0.010	1.00	325 ± 1.01	1810 ± 6
	Trifluralin	1.01 ± 0.02	685 ± 100	0.98	14.6 ± 0.88	0.055 ± 0.004	0.99	770 ± 2.58	4290 ± 14
OR3	Atrazine	0.53 ± 0.06	358 ± 68	0.93	6.37 ± 0.20	0.037 ± 0.009	0.95	26.6 ± 6.62	104 ± 26
	Chlorfenvinphos	0.57 ± 0.00	612 ± 124	0.99	5.44 ± 0.32	0.181 ± 0.059	0.97	61.3 ± 16.8	241 ± 63
	Endosulfan sulfate	0.70 ± 0.05	2021 ± 162	0.97	15.6 ± 5.91	0.217 ± 0.078	0.99	555 ± 187	2184 ± 791
	Trifluralin	0.71 ± 0.00	3660 ± 269	0.98	17.0 ± 1.54	0.402 ± 0.090	0.99	1973 ± 522	7764 ± 2054
OR4	Atrazine	0.39 ± 0.03	723 ± 26.3	0.91	6.19 ± 0.36	0.12 ± 0.02	0.95	24.5 ± 2.3	96.4 ± 11.8
	Chlorfenvinphos	0.65 ± 0.05	566 ± 68	0.96	5.67 ± 0.12	0.17 ± 0.04	0.97	88.4 ± 2.8	348 ± 11
	Endosulfan sulfate	0.70 ± 0.04	2023 ± 167	0.97	15.6 ± 4.1	0.22 ± 0.05	0.99	596 ± 167	2344 ± 486
	Trifluralin	0.71 ± 0.02	3693 ± 122	0.98	27.1 ± 4.6	0.26 ± 0.06	0.99	2093 ± 394	8232 ± 1551

Footnotes: n_f_: Freundlich coefficient correlated with adsorption intensity, K_f_ (L Kg^−1^): Freundlich constant correlated with the maximum multilayer adsorption capacity, Q_m_ (µg g^−1^): Langmuir constant representing the maximum sorption capacity relative to the total surface coverage, K: Langmuir constant representing the enthalpy of sorption, K_d_: lineal sorption constant, calculated for one sorption concentration (200 µg L^−1^), K_oc_: normalized organic carbon coefficient, calculated as (K_d_/%COT) · 100, where K_d_ = Q_e_/C_e_.

**Table 4:** Desorption parameters (OR1 = sawdust, OR2 = chicken manure, OR3 = orujillo, OR4 = composted urban solid waste).

		n_fd_	K_fd_ (L kg^−1^)	r^2^	H	%D
OR1	Atrazine	0.14 ± 0.01	2726 ± 188	0.91	498	40
	Chlorfenvinphos	1.98 ± 0.69	0.60 ± 0.33	0.58	36	100
	Endosulfan sulfate	0.20 ± 0.04	6628 ± 463	0.75	400	25
	Trifluralin	0.13 ± 0.01	9118 ± 610	0.96	630	18
OR2	Atrazine	0.13 ± 0.01	2287 ± 39	0.95	571	80
	Chlorfenvinphos	(a)	(a)	(a)	(a)	100
	Endosulfan sulfate	0.24 ± 0.03	5472 ± 612	0.94	387	28
	Trifluralin	0.08 ± 0.01	11,844 ± 286	0.91	1236	11
OR3	Atrazine	0.028 ± 0.01	3449 ± 290	0.93	1783	15
	Chlorfenvinphos	(a)	(a)	(a)	(a)	100
	Endosulfan sulfate	0.083 ± 0.002	9562 ± 1236	0.74	680	25
	Trifluralin	0.017 ± 0.005	15,312 ± 134	0.79	4201	4
OR4	Atrazine	0.14 ± 0.05	1542 ± 542	0.73	258	60
	Chlorfenvinphos	(a)	(a)	(a)	(a)	103
	Endosulfan sulfate	0.08 ± 0.01	10,645 ± 1401	0.62	861	19
	Trifluralin	0.02 ± 0.00	15,350 ± 113	0.77	4164	4

Footnotes: (a) Calculation was not possible. n_fd_: Freundlich coefficient correlated with desorption intensity. K_fd_ (L Kg^−1^): Freundlich constant correlated with the maximum multilayer desorption capacity. H: hysteresis coefficient. % D: desorption percentages.

## Data Availability

The data presented in this study are available on request from the corresponding author. The data are not publicly available due to privacy reasons.

## Supplementary Materials

The following supporting information can be downloaded at: https://www.mdpi.com/article/10.3390/toxics10020085/s1: Table S1: physicochemical properties of the studied pesticides. Table S2. Correlation coefficients between sorption-desorption and kinetic parameters and pesticides physicochemical properties. Table S3. Determination coefficients between sorption-desorption and kinetic parameters and physicochemical properties of organic wastes. Figure S1: Weber–Morris intraparticle diffusion model of each studied adsorbent for the different pesticides. Figure S2: Freundlich sorption isotherms of all pesticides on each organic residue. Figure S3: Sorption—desorption isotherms trifluralin on each organic residue. Figure S4: Sorption—desorption isotherms atrazine on each organic residue. Figure S5: Sorption—desorption isotherms chlorfenvinphos on each organic residue. Figure S6: Sorption—desorption isotherms endosulfan sulfate on each organic residue.
