# Supplementary material for: Sorption/Desorption and Kinetics of Atrazine, Chlorfenvinphos, Endosulfan Sulfate and Trifluralin on Agro-Industrial and Composted Organic Wastes
Source: Toxics. 2022 Feb 14;10(2):85. doi: 10.3390/toxics10020085 (PMC8877077; doi:10.3390/toxics10020085)
Supplement: Supplementary file 1 [file toxics-10-00085-s001.zip › toxics-1554381-supplementary.pdf]

toxics

Article

Sorption/Desorption and Kinetics of Atrazine, Chlorfenvinphos, Endosulfan Sulfate and Trifluralin on Agro-Industrial and Composted Organic Wastes

Raquel Rojas [1,2], Guillermo Repetto [1,*], José Morillo [2] and José Usero [2]

[1] Area of Toxicology, Department of Molecular Biology and Biochemical Engineering, Universidad Pablo de Olavide, Ctra. de Utrera Km 1, 41013 Seville, Spain; rrojrod@upo.es
[2] Department of Chemical and Environmental Engineering, University of Seville, Camino de los Descubrimientos s/n, 41092 Seville, Spain; jmorillo@us.es (J.M.); usero@us.es (J.U.)
* Correspondence: grepkuh@upo.es

Citation: Rojas, R.; Repetto, G.; Morillo, J.; Usero, J. Sorption/Desorption and Kinetics of Atrazine, Chlorfenvinphos, Endosulfan Sulfate and Trifluralin on Agro-Industrial and Composted Organic Wastes. *Toxics* **2022**, *10*, 85. https://doi.org/10.3390/10.3390/toxics10020085

Academic Editor: Xin-Gui Li

Received: 29 December 2021
Accepted: 9 February 2022
Published: 14 February 2022

Publisher's Note: MDPI stays neutral with regard to jurisdictional claims in published maps and institutional affiliations.

Copyright: © 2022 by the authors. Submitted for possible open access publication under the terms and conditions of the Creative Commons Attribution (CC BY) license (https://creativecommons.org/licenses/by/4.0/).

Table S1. physicochemical properties of the studied pesticides.

Pesticide	Type	2D structure diagram	Chem. Family	Chem. Formula	Molecular Weight (mol^{-1})	S_w (20 °C mg L^{-1})	DT50 typical (days)	GUS	K_{oc} (mL g^{-1})	Log K_{ow}
Atrazine	Herbicide	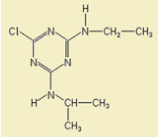	Triazine	$C_8H_{14}ClN_5$	215.68	35	75	3.20	100	2.75
Endosulfan sulfate	Metabolite	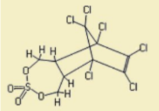	Organochlorine	$C_9H_6Cl_6O_4S$	422.93	0.48	-	-	5194	3.66
Chlorfenvinphos	Insecticid, Acaricide	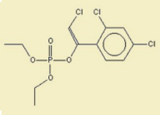	Organophosfate	$C_{12}H_{14}Cl_3O_{49}$	359.57	145	40	1.87	680	3.85
Trifluralin	Herbicide	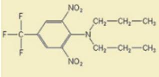	Dinitroaniline	$C_{13}H_{16}F_3N_3O_4$	335.28	0.221	181	0.13	15800	5.27

Footnotes: S_w (20 °C mg L^{-1}): Water solubility. DT50: typical half life. GUS: GUS indexes coefficient. K_{oc}: normalized organic carbon coefficient. K_{ow}: octanol/water coefficient. Data from PPDB (2020).

Table S2. Correlation coefficients between sorption-desorption and kinetic parameters and pesticides physicochemical properties ($n = 4$, $p < 0.05$).

	Molecular Weight (mol^{-1})	S_w (20 °C mg/L)	DT50 typical(days)	GUS	K_{oc} (mL/g)	Log K_{ow}	Molecular Weight (mol^{-1})	S_w (20 ºC mg/L)	DT50 typical(days)	GUS	K_{oc} (mL/g)	Log K_{ow}
	Sorption capacity (%) (600 mg - Effect of adsorbent dose)						K_2					
OR1	0.89	0.44	−0.22	−0.64	0.31	0.57	0.05	−0.45	0.97	−0.48	0.42	0.68
OR2	0.85	−0.11	0.37	−0.93	0.70	0.95	0.46	−0.66	0.77	−0.84	0.84	0.93
OR3	0.88	−0.08	0.31	−0.93	0.70	0.94	0.48	−0.83	0.58	−0.84	0.96	0.85
OR4	0.82	−0.36	0.44	−0.99	0.86	0.99	0.06	−0.42	0.97	−0.49	0.41	0.68
	K_i						X_i					
OR1	0.14	0.85	−0.90	0.38	−0.55	−0.52	0.89	−0.01	0.27	−0.91	0.65	0.91
OR2	−0.53	0.79	−0.58	0.87	−0.96	−0.89	0.83	−0.27	0.42	−0.97	0.81	0.99
OR3	0.06	1.00	−0.61	0.40	−0.69	−0.44	0.80	−0.37	0.46	−0.98	0.86	1.00
OR4	0.28	0.81	−0.91	0.24	−0.42	−0.40	0.67	−0.50	0.62	−0.95	0.87	0.99
	N_{fa}						K_{fa}					
OR1	0.04	−0.93	0.80	−0.53	0.73	0.62	0.76	−0.62	0.27	−0.94	0.99	0.88
OR2	0.60	0.61	0.04	−0.41	0.00	0.45	0.36	−0.83	0.74	−0.78	0.88	0.84
OR3	0.72	−0.59	0.48	−0.97	0.96	0.97	0.42	−0.63	0.81	−0.81	0.79	0.91
OR4	0.92	−0.07	0.23	−0.94	0.72	0.92	0.32	−0.70	0.85	−0.75	0.77	0.86
	K_d						K_{oc}					
OR1	0.50	−0.75	0.67	−0.87	0.92	0.91	0.50	−0.75	0.67	−0.87	0.92	0.91
OR2	0.32	−0.64	0.87	−0.74	0.73	0.87	0.32	−0.64	0.87	−0.74	0.73	0.87
OR3	0.24	−0.57	0.92	−0.67	0.63	0.82	0.24	−0.57	0.92	−0.67	0.63	0.82
OR4	0.25	−0.57	0.92	−0.67	0.63	0.82	0.25	−0.57	0.92	−0.67	0.63	0.82
	N_{fd}						K_{fd}					
OR1	0.27	0.97	−0.50	0.16	−0.52	−0.19		−0.88	0.75	−0.71	0.85	0.77
OR2	0.56	−0.21	−0.85	−0.26	0.43	0.02	0.46	−0.76	0.87	−0.72	0.59	0.87
OR3	0.68	−0.36	−0.76	−0.41	0.56	0.17	0.63	−0.88	0.75	−0.85	0.74	0.95
OR4	−0.62	0.87	−0.76	0.84	−0.73	−0.95	0.75	−0.94	0.63	−0.92	0.84	0.99
	H						%D					
OR1	−0.33	−0.91	0.76	−0.17	0.45	0.28	0.05	1.00	−0.60	0.40	−0.70	−0.43
OR2	−0.06	−0.31	1.00	−0.26	0.08	0.49	−0.37	0.86	−0.68	0.78	−0.91	−0.82
OR3	−0.17	−0.21	0.99	−0.16	−0.02	0.39	0.31	0.95	−0.64	0.16	−0.49	−0.23
OR4	0.28	−0.62	0.95	−0.58	0.42	0.76	−0.20	0.95	−0.67	0.64	−0.85	−0.68

Footnotes: S_w (Water solubility), DT50 (half-life), GUS (GUS indexes coefficient), K_{oc} (normalized organic carbon coefficient) and K_{ow} (octanol/water coefficient), were taken from PPDB (2020).

Table S3. Determination coefficients between sorption-desorption and kinetic parameters and physicochemical properties of organic wastes ($p < 0.05$).

	%TOC	BET	pH	C	N	O	Na	Mg	Al	Si	P	S	Cl	K	Ca	Ti	Fe	Cu	
n	4	4	4	4	2	4	2	3	2	3	1	2	3	3	3	1	2	1	*n*
	Sorption capacity (%) (600 mg - Effect of adsorbent dose)																		
AT	0.52	−0.47	−0.92	**0.95**	n/a	−0.20	n/a	−0.39	n/a	−0.01	n/a	n/a	−0.52	0.49	−0.52	n/a	n/a	n/a	4
TF	−0.46	0.40	0.89	−0.93	n/a	0.27	n/a	0.04	n/a	−0.34	n/a	n/a	0.18	−0.15	0.19	n/a	n/a	n/a	4
CF	0.78	−0.87	**−0.97**	**0.95**	n/a	0.36	n/a	−0.99	n/a	−0.87	n/a	1.00	**−1.00**	**1.00**	**−1.00**	n/a	n/a	n/a	4
ES	0.03	0.33	0.75	−0.62	n/a	0.20	n/a	0.05	n/a	−0.33	n/a	n/a	0.20	−0.17	0.20	n/a	n/a	n/a	4
	Q_e exp																		
AT	0.42	−0.51	−0.94	0.92	n/a	−0.14	n/a	−0.35	n/a	0.03	n/a	n/a	−0.48	0.46	−0.49	n/a	n/a	n/a	4
TF	−0.24	0.38	0.87	−0.83	n/a	0.26	n/a	0.03	n/a	−0.35	n/a	n/a	0.18	−0.15	0.18	n/a	n/a	n/a	4
CF	0.86	−0.83	−0.94	**0.96**	n/a	0.32	n/a	−0.99	n/a	−0.97	n/a	n/a	−0.96	0.96	−0.95	n/a	n/a	n/a	4
ES	0.19	0.30	0.63	−0.46	n/a	0.12	n/a	0.09	n/a	−0.30	n/a	n/a	0.23	−0.20	0.24	n/a	n/a	n/a	4
	K_2																		
AT	0.86	−0.84	−0.42	0.43	n/a	0.84	n/a	−0.88	n/a	**−1.00**	n/a	n/a	−0.81	0.83	**	n/a	n/a	n/a	4
TF	0.66	**−0.96**	−0.89	0.79	n/a	0.60	n/a	−0.96	n/a	−0.78	n/a	n/a	−0.99	0.98	−0.99	n/a	n/a	n/a	4
CF	0.52	−0.71	−0.10	0.02	n/a	**0.98**	n/a	−0.95	n/a	**−1.00**	n/a	n/a	−0.90	0.91	−0.90	n/a	n/a	n/a	4
ES	0.67	−0.49	−0.90	**0.98**	n/a	−0.15	n/a	−0.95	n/a	**−1.00**	n/a	n/a	−0.90	0.91	−0.90	n/a	n/a	n/a	4
	K_i																		
AT	**−0.98**	0.83	0.62	−0.68	n/a	−0.62	n/a	0.83	n/a	0.98	n/a	n/a	0.74	−0.76	0.74	n/a	n/a	n/a	4
TF	−0.10	−0.56	−0.56	0.31	n/a	0.36	n/a	−0.50	n/a	−0.13	n/a	n/a	−0.62	0.60	−0.62	n/a	n/a	n/a	4
CF	−0.50	0.91	0.87	−0.72	n/a	−0.55	n/a	0.87	n/a	0.62	n/a	n/a	0.93	−0.92	0.93	n/a	n/a	n/a	4
ES	−0.33	0.88	0.61	−0.40	n/a	−0.77	n/a	0.88	n/a	0.64	n/a	n/a	0.94	−0.93	0.94	n/a	n/a	n/a	4
	X_i																		
AT	0.91	−0.90	−0.88	0.89	n/a	0.49	n/a	−0.97	n/a	−0.99	n/a	n/a	−0.93	0.94	−0.93	n/a	n/a	n/a	4
TF	−0.02	−0.21	0.46	−0.56	n/a	0.78	n/a	−0.95	n/a	**−1.00**	n/a	n/a	−0.90	0.91	−0.89	n/a	n/a	n/a	4
CF	0.62	−0.89	**−0.96**	0.86	n/a	0.43	n/a	−0.90	n/a	−0.67	n/a	n/a	−0.96	0.95	−0.96	n/a	n/a	n/a	4
ES	0.44	−0.87	−0.38	0.21	n/a	**0.96**	n/a	−0.99	n/a	−0.87	n/a	n/a	**−1.00**	**1.00**	**−1.00**	n/a	n/a	n/a	4
	n_{fa}																		
AT	0.03	−0.51	−0.77	0.60	n/a	0.06	n/a	−0.33	n/a	0.05	n/a	n/a	−0.47	0.44	−0.47	n/a	n/a	n/a	4
TF	−0.27	−0.21	−0.56	0.39	n/a	−0.19	n/a	0.00	n/a	0.38	n/a	n/a	−0.14	0.11	−0.15	n/a	n/a	n/a	4
CF	−0.77	0.17	0.09	−0.31	n/a	−0.12	n/a	0.13	n/a	0.50	n/a	n/a	−0.02	−0.01	−0.02	n/a	n/a	n/a	4
ES	−0.35	−0.18	−0.49	0.30	n/a	−0.16	n/a	−0.01	n/a	0.37	n/a	n/a	−0.15	0.13	−0.16	n/a	n/a	n/a	4
	K_{fa}																		
AT	−0.28	0.73	0.88	−0.71	n/a	−0.28	n/a	0.62	n/a	0.27	n/a	n/a	0.72	−0.70	0.73	n/a	n/a	n/a	4
TF	0.09	0.29	0.71	−0.58	n/a	0.22	n/a	0.01	n/a	−0.37	n/a	n/a	0.15	−0.12	0.16	n/a	n/a	n/a	4
CF	0.34	0.14	0.50	−0.33	n/a	0.24	n/a	−0.07	n/a	−0.44	n/a	n/a	0.07	−0.05	0.08	n/a	n/a	n/a	4
ES	0.22	0.24	0.61	−0.45	n/a	0.20	n/a	0.00	n/a	−0.38	n/a	n/a	0.14	−0.12	0.15	n/a	n/a	n/a	4
	K_d																		
AT	0.43	−0.50	−0.94	0.92	n/a	−0.16	n/a	−0.34	n/a	0.05	n/a	n/a	−0.47	0.44	−0.47	n/a	n/a	n/a	4
TF	−0.04	0.37	0.79	−0.68	n/a	0.19	n/a	0.08	n/a	−0.30	n/a	n/a	0.22	−0.20	0.23	n/a	n/a	n/a	4
CF	−0.17	0.65	0.84	−0.66	n/a	−0.20	n/a	0.51	n/a	0.14	n/a	n/a	0.63	−0.61	0.63	n/a	n/a	n/a	4
ES	0.12	0.35	0.68	−0.51	n/a	0.09	n/a	0.14	n/a	−0.25	n/a	n/a	0.28	−0.25	0.29	n/a	n/a	n/a	4
	K_{oc}																		
AT	−0.74	0.09	0.24	−0.49	n/a	0.15	n/a	−0.10	n/a	0.29	n/a	n/a	−0.24	0.21	−0.25	n/a	n/a	n/a	4
TF	−0.25	0.42	0.88	−0.83	n/a	0.22	n/a	0.11	n/a	−0.28	n/a	n/a	0.25	−0.22	0.26	n/a	n/a	n/a	4
CF	−0.49	0.70	**0.98**	−0.92	n/a	−0.10	n/a	0.69	n/a	0.36	n/a	n/a	0.78	−0.77	0.79	n/a	n/a	n/a	4
ES	−0.45	0.47	0.93	−0.93	n/a	0.19	n/a	0.29	n/a	−0.09	n/a	n/a	0.43	−0.40	0.43	n/a	n/a	n/a	4
	n_{fd}																		
AT	−0.49	0.60	−0.04	0.09	n/a	−0.94	n/a	0.90	n/a	**1.00**	n/a	n/a	0.83	−0.85	0.83	n/a	n/a	n/a	4
TF	0.20	−0.36	−0.85	0.81	n/a	−0.27	n/a	0.00	n/a	0.38	n/a	n/a	−0.14	0.11	−0.15	n/a	n/a	n/a	4
CF	n/a	n/a	n/a	n/a	n/a	n/a	n/a	n/a	n/a	n/a	n/a	n/a	n/a	n/a	n/a	n/a	n/a	n/a	2

ES	−0.27	−0.21	−0.57	0.39	n/a	−0.19	n/a	0.00	n/a	0.38	n/a	n/a	−0.14	0.11	−0.15	n/a	n/a	n/a	4
	K_{fd}																		
AT	0.75	**−0.96**	−0.57	0.49	n/a	0.90	n/a	−0.99	n/a	−0.96	n/a	n/a	−0.96	0.97	−0.96	n/a	n/a	n/a	4
TF	−0.19	0.37	0.85	−0.80	n/a	0.26	n/a	0.01	n/a	−0.37	n/a	n/a	0.15	−0.12	0.16	n/a	n/a	n/a	4
CF	n/a	n/a	n/a	n/a	n/a	n/a	n/a	n/a	n/a	n/a	n/a	n/a	n/a	n/a	n/a	n/a	n/a	n/a	2
ES	0.16	0.38	0.64	−0.45	n/a	0.01	n/a	0.20	n/a	−0.19	n/a	n/a	0.34	−0.31	0.34	n/a	n/a	n/a	4
	H																		
AT	0.55	−0.71	−0.10	0.03	n/a	**0.98**	n/a	−0.95	n/a	**−1.00**	n/a	n/a	−0.89	0.90	−0.89	n/a	n/a	n/a	4
TF	0.01	0.30	0.76	−0.65	n/a	0.25	n/a	−0.01	n/a	−0.39	n/a	n/a	0.13	−0.10	0.14	n/a	n/a	n/a	4
CF	n/a	n/a	n/a	n/a	n/a	n/a	n/a	n/a	n/a	n/a	n/a	n/a	n/a	n/a	n/a	n/a	n/a	n/a	2
ES	−0.06	0.55	0.79	−0.61	n/a	−0.10	n/a	0.38	n/a	0.00	n/a	n/a	0.51	−0.48	0.51	n/a	n/a	n/a	4
	%D																		
AT	−0.91	0.65	0.31	−0.41	n/a	−0.65	n/a	0.68	n/a	0.91	n/a	n/a	0.57	−0.59	0.56	n/a	n/a	n/a	4
TF	0.22	−0.37	−0.86	0.82	n/a	−0.27	n/a	0.00	n/a	0.38	n/a	n/a	−0.14	0.12	−0.15	n/a	n/a	n/a	4
CF	n/a	n/a	n/a	n/a	n/a	n/a	n/a	n/a	n/a	n/a	n/a	n/a	n/a	n/a	n/a	n/a	n/a	n/a	2
ES	0.03	−0.69	−0.55	0.30	n/a	0.56	n/a	−0.67	n/a	−0.34	n/a	n/a	−0.77	0.75	−0.78	n/a	n/a	n/a	4

Footnotes: AT: Atrazine, TF: Trifluralin, CF: chlorfenvinphos, ES: endosulfan sulfate. Kinetic parameters: Q_e exp (quantity of pesticide adsorbed at equilibrium), K_2 (Ho and McKay rate constants), K_i (intraparticle diffusion rate constant), X_i (Morris-Weber constant, proportional to the boundary layer thickness). Sorption constants: n_{fa} (Freundlich coefficient correlated to adsorption intensity), K_{fa} (Freundlich constant correlated to the maximum multilayer adsorption capacity). K_d (lineal sorption constant, calculated for one sorption concentration (200 μg L^{-1})), K_{oc} (normalized organic carbon coefficient, calculated as (K_d/%COT) · 100, where $K_d = Q_e/C_e$), n_{fd} (Freundlich coefficient correlated to desorption intensity) Desorption constants: K_{fd} (Freundlich constant correlated to the maximum multilayer desorption capacity), H (hysteresis coefficient), %D (desorption percentages). In bold: significant correlations.

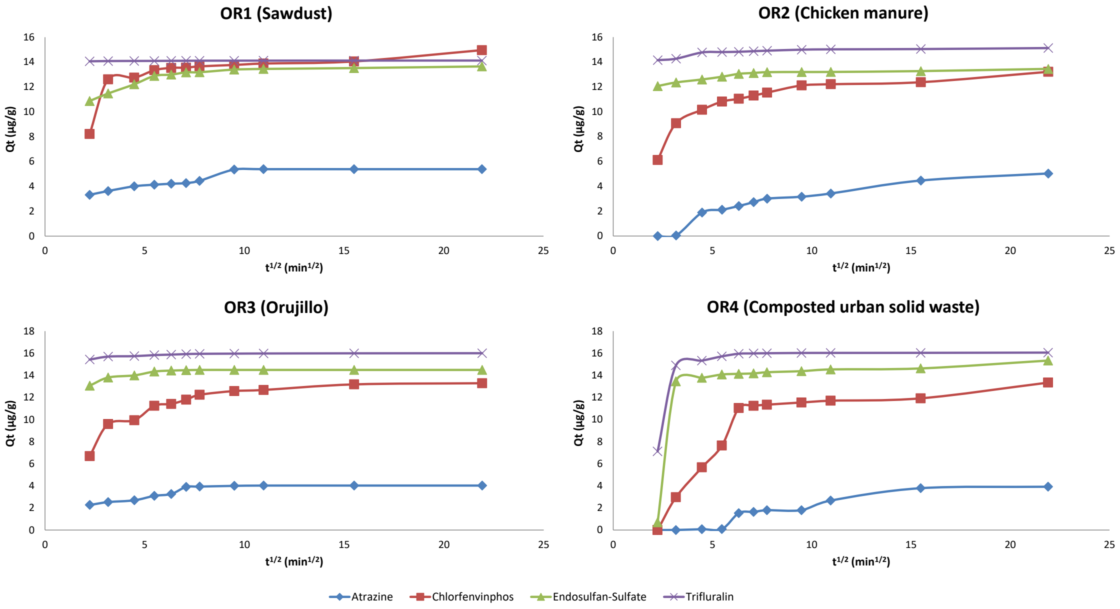

Figure S1. Weber-Morris intraparticle diffusion model of each studied adsorbent for the different pesticides.

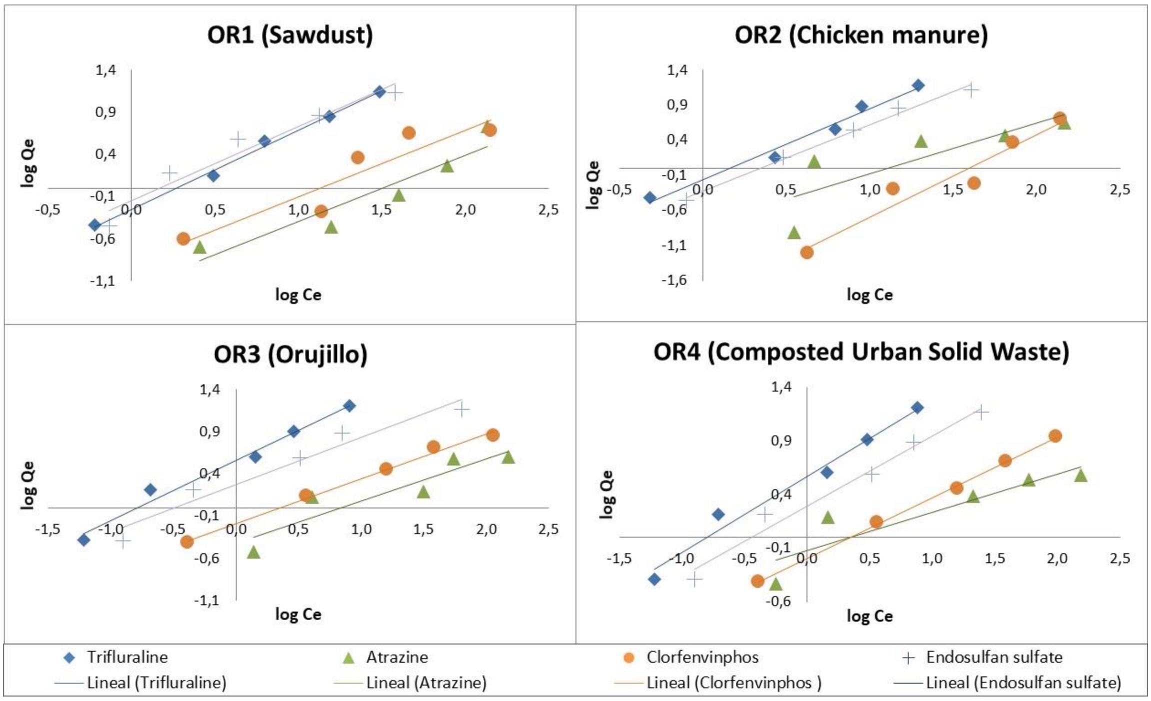

Figure S2. Freundlich sorption isotherms of all pesticides on each organic residue.

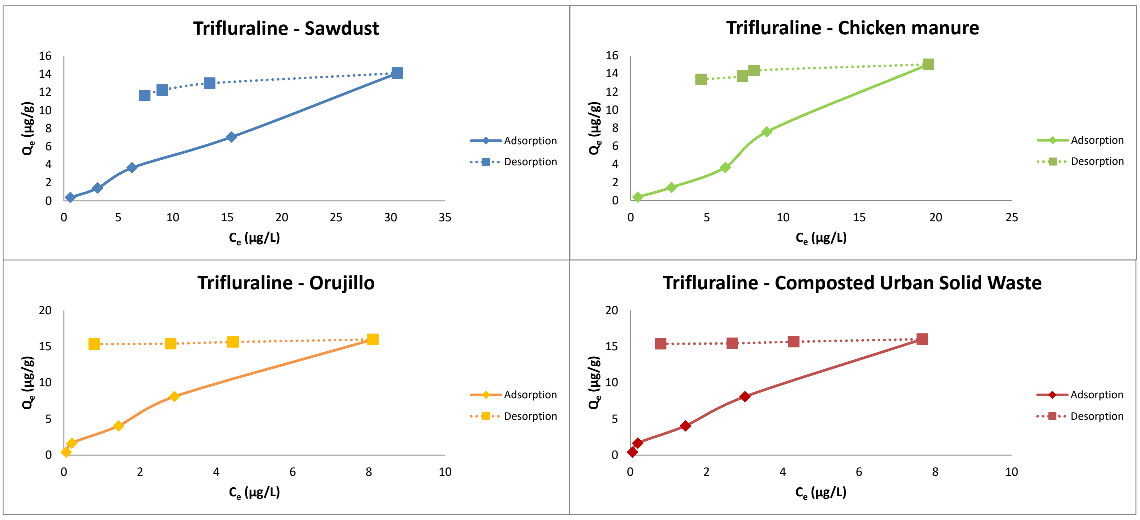

Figure S3. Sorption - desorption isotherms trifluralin on each organic residue.

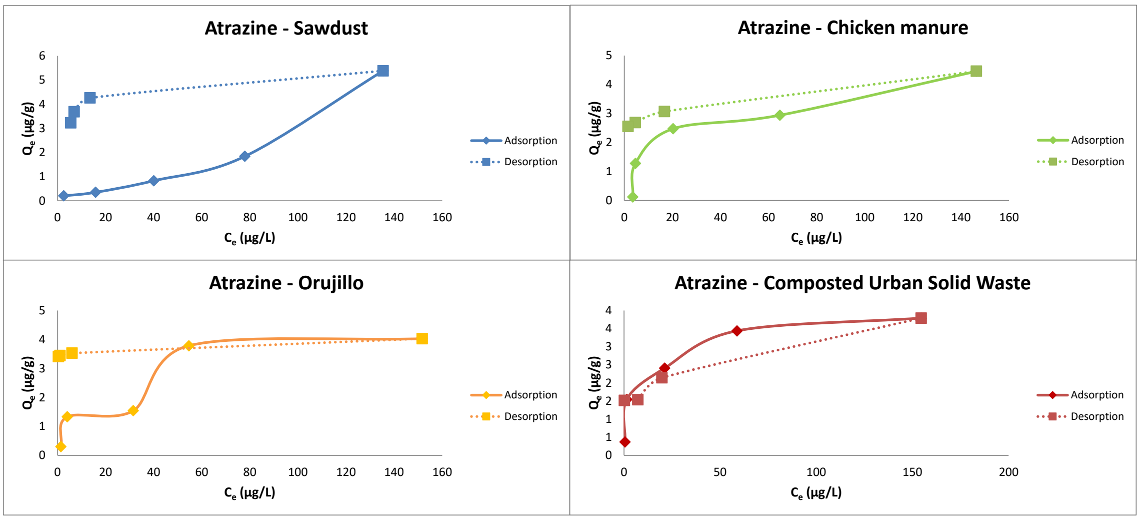

Figure S4. Sorption - desorption isotherms atrazine on each organic residue.

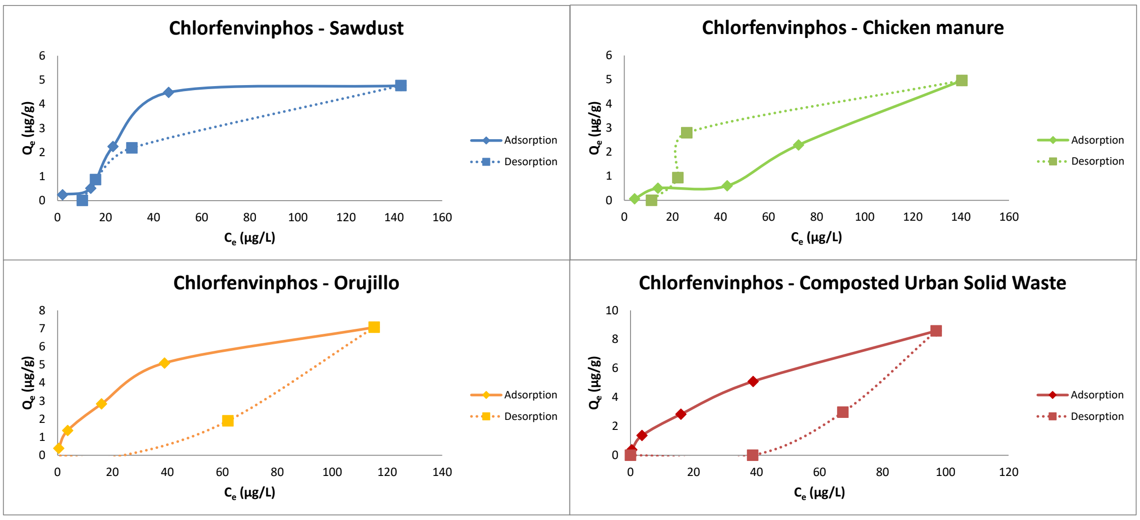

Figure S5. Sorption - desorption isotherms chlorfenvinphos on each organic residue.

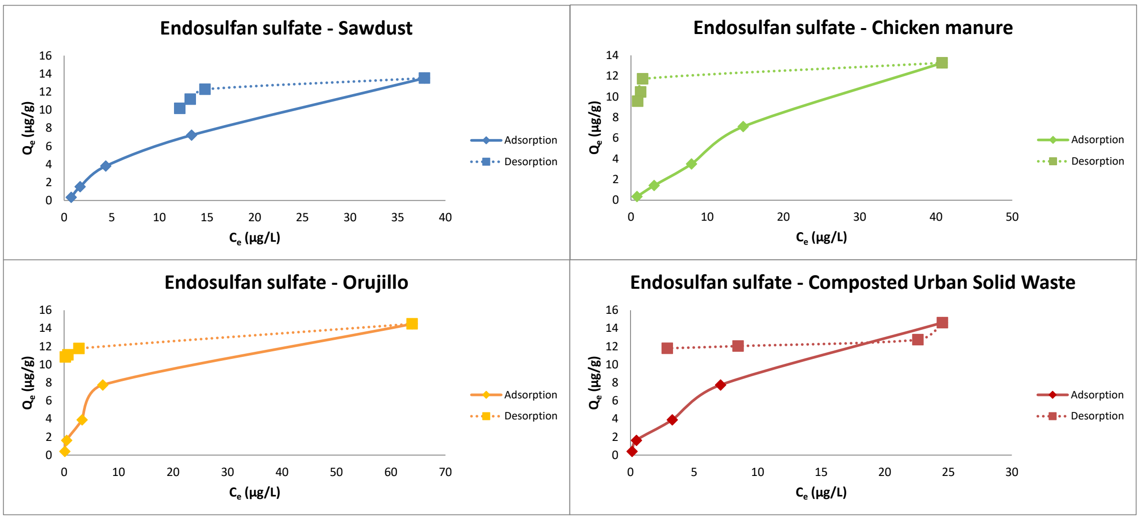

Figure S6. Sorption - desorption isotherms endosulfan sulfate on each organic residue.
